# Exploring the Role of Gut Microbiota and Probiotics in Acute Pancreatitis: A Comprehensive Review

**DOI:** 10.3390/ijms26073433

**Published:** 2025-04-06

**Authors:** Enrico Celestino Nista, Simone Parello, Mattia Brigida, Giulio Amadei, Angela Saviano, Sara Sofia De Lucia, Carmine Petruzziello, Alessio Migneco, Veronica Ojetti

**Affiliations:** 1 Fondazione Policlinico Gemelli, Istituiti di Ricovero e Cura a Carattere Scientifico (IRCCS), 00168 Rome, Italy; enricocelestino.nista@policlinicogemelli.it (E.C.N.); simone.parello@gmail.com (S.P.); a.giulio97@gmail.com (G.A.); angela.saviano@policlinicogemelli.it (A.S.); saras.delucia@gmail.com (S.S.D.L.); alessio.migneco@policlinicogemelli.it (A.M.); 2 Gastroenterology Unit, Policlinico Universitario Tor Vergata, 00133 Rome, Italy; mattiabrigida@hotmail.com; 3 Ospedale San Carlo di Nancy, GVM Research, 00165 Rome, Italy; 4 Department of Internal Medicine, UniCamillus International Medical University of Rome, 00131 Rome, Italy

**Keywords:** acute pancreatitis, gut microbiota, probiotics, gut-pancreas axis

## Abstract

Acute pancreatitis (AP) is a common and potentially severe gastrointestinal condition characterized by acute inflammation of the pancreas. The pathophysiology of AP is multifactorial and intricate, involving a cascade of events that lead to pancreatic injury and systemic inflammation. The progression of AP is influenced by many factors, including genetic predispositions, environmental triggers, and immune dysregulation. Recent studies showed a critical involvement of the gut microbiota in shaping the immune response and modulating inflammatory processes during AP. This review aims to provide a comprehensive overview of the emerging role of gut microbiota and probiotics in AP. We analyzed the implication of gut microbiota in pathogenesis of AP and the modification during an acute attack. The primary goals of microbiome-based therapies, which include probiotics, prebiotics, antibiotics, fecal microbiota transplantation, and enteral nutrition, are to alter the composition of the gut microbial community and the amount of metabolites derived from the microbiota. By resetting the entire flora or supplementing it with certain beneficial organisms and their byproducts, these therapeutic approaches aim to eradicate harmful microorganisms, reducing inflammation and avoiding bacterial translocation and the potential microbiota-based therapeutic target for AP from nutrition to pre- and probiotic supplementation to fecal transplantation.

## 1. Introduction

Acute pancreatitis (AP) is a prevalent and potentially severe gastrointestinal condition characterized by acute inflammation of the pancreas. Globally, AP accounts for a significant number of hospital admissions, with incidence rates varying between 13 and 45 cases per 100,000 individuals annually [1]. While the majority of cases present as mild and resolve with supportive care, approximately 20–30% progress to severe forms, often accompanied by systemic complications such as multi-organ dysfunction syndrome (MODS), pancreatic necrosis, and sepsis [2]. This disease burden, compounded by a lack of targeted pharmacological therapies, highlights the pressing need for novel insights into its pathogenesis and innovative therapeutic strategies.

The pathophysiology of AP is multifactorial and intricate, involving a cascade of events that lead to pancreatic injury and systemic inflammation [3]. It is initiated by the premature activation of pancreatic enzymes, particularly trypsin, within the acinar cells. This triggers a localized inflammatory response that can rapidly escalate into a systemic inflammatory response syndrome (SIRS). The progression of AP is influenced by numerous factors, including genetic predispositions, environmental triggers, and immune dysregulation [4].

Central to this process is the interaction between the pancreas and other organ systems, notably the gastrointestinal tract. The gut plays a dual role in AP, serving as both a contributor to disease progression and a potential therapeutic target. Recent research has unveiled the critical involvement of the gut microbiota—a complex and diverse community of microorganisms residing in the gastrointestinal tract—in shaping the immune response and modulating inflammatory processes during AP [5].

The human gut harbors trillions of microorganisms, including bacteria, archaea, fungi, and viruses, collectively referred to as the gut microbiota. These microorganisms play an essential role in maintaining intestinal homeostasis, regulating host metabolism, supporting the immune system, and protecting against pathogenic invasion [6]. However, during AP, the homeostatic balance of the gut microbiota is often disrupted, leading to a state of dysbiosis.

Dysbiosis in AP has been linked to several pathological mechanisms, including the following: (1) increased intestinal permeability: the loss of tight junction integrity in the intestinal epithelium facilitates bacterial translocation into the bloodstream, contributing to systemic inflammation and infection [7]; (2) enhanced pro-inflammatory signaling: dysbiosis promotes an imbalance in immune responses, favoring the production of pro-inflammatory cytokines such as interleukin (IL)−6, IL−1β, and Tumor necrosis factor (TNF)-α [8]; and (3) depletion of beneficial microbial metabolites: reduced levels of short-chain fatty acids (SCFAs) and other microbial-derived molecules impair intestinal and systemic immune regulation, exacerbating inflammation [9].

The interplay between the gut microbiota and the pancreas, often referred to as the gut–pancreas axis, involves bidirectional signaling mediated by microbial metabolites, inflammatory mediators, and neuroendocrine pathways. Understanding the mechanisms underlying this axis is critical for identifying new therapeutic targets [10].

Probiotics, defined as live microorganisms that confer health benefits to the host when administered in adequate amounts, have garnered significant attention for their potential role in modulating gut microbiota and mitigating inflammation. In the context of AP, probiotics may exert multiple beneficial effects, including restoration of gut microbial balance, strengthening of the intestinal barrier, modulation of systemic and local immune responses, and reduction of bacterial translocation and systemic infection risk [11].

Experimental studies in animal models of AP have demonstrated that probiotics can reduce inflammatory markers, improve gut barrier function, and decrease the severity of pancreatic injury [12]. However, clinical studies have yielded mixed results. While some trials suggest probiotics may reduce the incidence of infectious complications and hospital stay, others, such as the landmark PROPATRIA trial, reported no significant benefit and raised safety concerns [13]. These divergent findings underscore the complexity of microbiota-targeted therapies and highlight the importance of strain-specific effects, disease severity, and patient-specific factors in determining therapeutic outcomes.

Over the last years, many advanced approaches have been proposed for the study and management of pancreatic diseases, among them the following: multi-omics (in particular, metagenomics, metabolomics, and transcriptomics) [14], the use of genetically modified murine models [15,16], and the integration of artificial intelligence [17,18] for prediction of clinical evolution and of therapeutic response. Particularly, the growing interest in the role of gut microbiota in acute pancreatitis has led to the identification of specific patterns of dysbiosis associated with disease severity and systemic inflammation.

Moreover, not only has the microbiota a role in acute and chronic inflammation mechanisms but it has also been suggested that it might influence the progression of pancreatic cancer [19]. Indeed, there is evidence in the literature of a reduced abundance of beneficial microorganisms compared to in healthy individuals, and it has been postulated that this can promote the development of cancer cells [20]. For instance, subjects with higher Bifidobacterium levels in their gut microbiome have a lower risk of developing pancreatic cancer [21] and this has been linked to the capability of this bacterium to maintain a balanced gut microbiome and reducing inflammation [22], with mechanisms that will be further described within the review.

Gut microbiota might also directly or indirectly account for development of drug-resistance during the course of pancreatic cancer treatment [20], via the ability of some bacteria to absorb and metabolized some antineoplastic drugs, thus reducing their efficacy [23].

This review aims to provide a comprehensive overview of the emerging role of gut microbiota and probiotics in AP.

In the present study, the selection of literature was carried out through a systematic search of the main databases (PubMed, Scopus, Web of Science) using targeted keywords (e.g., “acute pancreatitis”, “gut microbiota”, “probiotics”, “intestinal dysbiosis”). Articles published in the past 10 years were included, with particular attention to clinical studies, meta-analyses, and systematic reviews. The content analysis was conducted critically, with the aim of highlighting the main pathogenetic mechanisms, experimental evidence, and emerging therapeutic perspectives.

We will explore the fundamental biology of the gut microbiota and its relationship with pancreatic health, mechanisms by which dysbiosis contributes to the pathogenesis and progression of AP, the evidence base for probiotics as a therapeutic intervention, including insights from preclinical and clinical studies, and current challenges and future directions for microbiota-targeted therapies in AP, including the development of personalized approaches and the integration of advanced technologies such as metagenomics and metabolomics.

By synthesizing existing knowledge and identifying critical gaps, this review seeks to advance our understanding of the gut–pancreas axis and its implications for AP management.

## 2. Microbiota Modification During Acute Pancreatis (AP)

**Figure 1:**
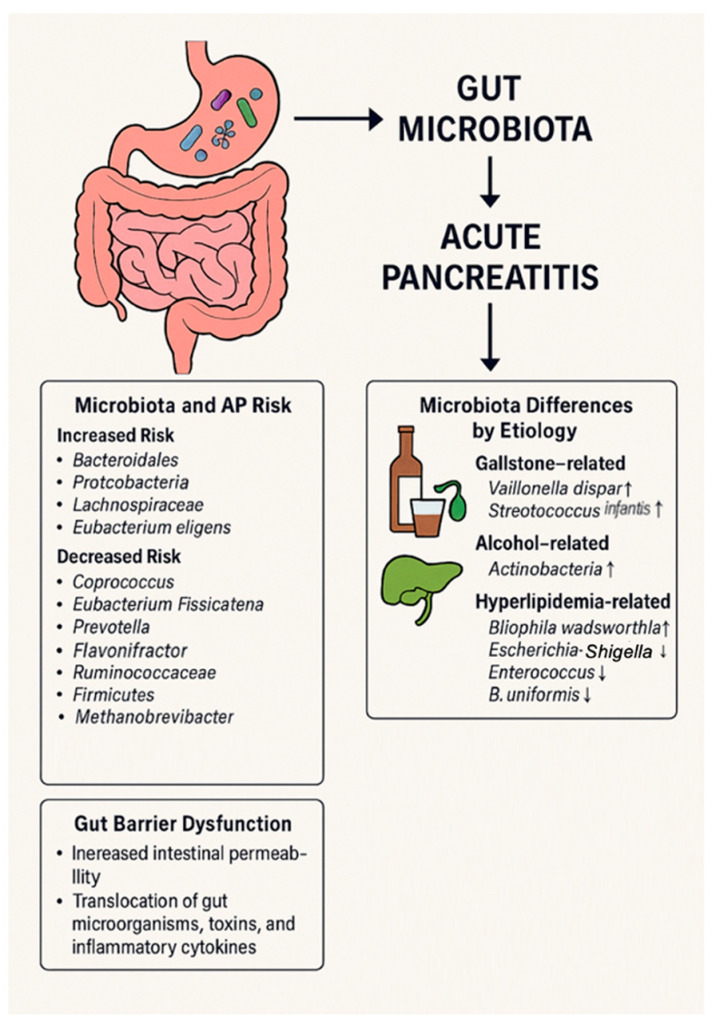
Gut barrier dysfunction mechanisms and gut microbiota alterations seen in acute pancreatitis.

In the field of microbiota research, there is growing interest in the interaction between gut microbiota and pancreatic disease [24]. Researchers are focused on how alterations of gut microbiome contribute to the development of AP and their potential association with disease severity. In the setting of AP, abnormal gut microbiota overgrowth occurs as a result of exocrine pancreatic insufficiency and decreased levels of antimicrobial peptides. Concurrently, the migration of intestinal microbiota exerts an influence on the pancreatic microenvironment, contributing to the severity of acute pancreatitis and amplifying the systemic inflammatory response [24,25]. In fact, the pancreas contributes to the body’s innate defense mechanisms. Notably, approximately 10% of the proteins in pancreatic juice consist of antimicrobial peptides, which play a key role in modulating the gut microbiome [26,27,28,29,30]. Ahuja et al. demonstrated that antimicrobials secreted by pancreatic acinar cells play a crucial role in shaping the gut microbiome, which is essential for maintaining intestinal innate immunity, barrier integrity, and overall survival [31]. This regulatory function is closely linked to calcium (Ca^2+^) signaling pathways, particularly those mediated by the Orai1 calcium channel. Orai1 is pivotal in modulating intracellular calcium dynamics, which are critical for immune cell activation and acinar cell function [31]. Supporting this, the deletion of the Orai1 Ca^2+^ channel in pancreatic acinar cells of adult mice resulted in intestinal bacterial overgrowth, microbiota dysbiosis, and increased mortality, underscoring its essential role in maintaining intestinal homeostasis [31]. Disruption of the gut microbiome further facilitates bacterial translocation to systemic circulation, triggering widespread inflammation. In this context, while supplementation with digestive enzymes proved ineffective, interventions that restricted bacterial overgrowth were linked to improved survival outcomes [31]. These findings highlight the essential role of pancreatic antimicrobial peptides in maintaining gut integrity, regulating the microbiome, and protecting against dysbiosis. This interplay ensures a balanced microbiota, which is crucial for preserving the structural and functional integrity of the intestinal epithelium and protecting against microbial dysbiosis. When this balance is disrupted, as observed in severe acute pancreatitis (SAP), it leads to an increase in intestinal permeability, allowing bacterial endotoxins to translocate into the bloodstream. Plasma endotoxin levels and serum levels of TNF-α, IL−6, IL−1 (pro-inflammatory cytokines) were significantly elevated in the SAP group compared to both the mild acute pancreatitis (MAP) group and the control group [32,33,34]. These cytokines are produced at the mucosal interface as a result of interactions between the gut microbiota and the intestinal immune system. Their dysregulation may contribute significantly to the development of systemic complications in AP, as suggested by prior studies [32,33,34]. Supporting these observations, a prospective clinical study by Tan et al. demonstrated distinct gut microbiota alterations in SAP patients [35]. Specifically, SAP patients exhibited increased *Enterococcus* levels and reduced *Bifidobacterium* levels compared to MAP patients. Moreover, serum IL−6 levels were positively correlated with Enterobacteriaceae and *Enterococcus* abundance, while showing a negative correlation with *Bifidobacterium*. Plasma endotoxin levels also exhibited a positive correlation with *Enterococcus* [35]. These findings highlight a potential mechanistic link between gut microbiota composition, endotoxin release, cytokine responses, and the severity of AP. In agreement with these findings, other studies have reported significant microbiota shifts in patients with AP. Specifically, an increased abundance of Bacteroidetes, Proteobacteria, *Escherichia–Shigella*, Erysipelotrichaceae, *Streptococcus*, and *Enterococcus* was observed, while the levels of Firmicutes and Actinobacteria were reduced [36,37,38]. Zhang et al. further corroborated these findings, demonstrating that fecal samples from AP patients showed a higher prevalence of Bacteroidetes and Proteobacteria and a decreased abundance of Firmicutes and Actinobacteria when compared to healthy volunteers [39]. These microbiota imbalances may play a pivotal role in disease pathophysiology. Specifically, the overrepresentation of certain bacterial taxa such as Bacteroidetes and Proteobacteria has been implicated in promoting bacterial translocation across the intestinal barrier [39]. Chen et al., in their study on rats with acute necrotizing pancreatitis, reported significant alterations in gut microbiota composition [40]. Specifically, they observed a marked decrease in the phyla Saccharibacteria and Tenericutes, alongside a notable increase in the genera *Escherichia–Shigella* and *Phascolarctobacterium*. Furthermore, taxa such as *Candidatus Saccharimonas*, Prevotellaceae, Lachnospiraceae, *Ruminiclostridium*, and *Ruminococcaceae* showed significant reductions, highlighting the profound microbiota disruptions associated with this condition [40]. These changes underline the significant alterations of gut microbiome associated with AP and their potential contribution to secondary infections and disease progression. In line with these observations, a more recent clinical study by Liu et al. further expanded on the microbiota alterations in AP patients, revealing reduced species and functional diversity compared to healthy individuals. Several bacterial species, including *Escherichia coli*, *Enterococcus*, *Parabacteroides*, and members of *Clostridium* and *Veillonella*, were found to be more abundant in AP patients [41]. Of particular concern is the predominance of *E. coli*, which has been identified as a high-risk pathogen in AP. This bacterium has been consistently linked to severe inflammatory gastrointestinal disorders such as Crohn’s disease, appendicitis, acute cholecystitis, and peritonitis [42,43,44,45,46]. Moreover, *E. coli* poses a significant risk for translocation from the gastrointestinal tract to extraintestinal sites, potentially leading to systemic infections [42]. Collectively, these findings suggest that *E. coli* plays a crucial role in the progression of AP and its complications, underscoring the importance of microbiota-targeted approaches in understanding and managing AP and related inflammatory disorders. While previous studies have focused on more severe forms of AP, the microbiota alterations in patients with MAP are less well understood. A study investigating the microbiota composition of the descending duodenum revealed subtle changes in patients with MAP [47]. The modest findings may be attributed to the comparison between healthy individuals and MAP patients. Alpha diversity showed no significant differences between the groups, but beta diversity analysis identified distinct microbiota profiles [47]. In MAP patients, the duodenal mucosa had higher levels of *Streptococcus* and *Neisseria* compared to controls, who exhibited a greater abundance of *Halothrix*. Additionally, *Actinobacillus* and *Oribacterium* were significantly more prevalent in the control group. Despite these microbiota differences, no notable inflammatory changes were observed in the duodenal mucosa of MAP patients [47]. A key finding of the study was the reduced expression of tight junction proteins (TJPs) in the duodenal mucosa of MAP patients, suggesting mild duodenal barrier dysfunction and subtle microbiota alterations. Although the sample size was limited, the study’s focus on descending duodenum samples provided unique insights into the impact of MAP on the intestinal environment [47]. The findings suggest that even in the absence of overt inflammation, dysregulation of gut microbiota composition and intestinal barrier function may contribute to the disease’s pathogenesis. Table 1 summarizes the microbiota alterations observed in AP patients. Figure 1 summarizes key data and outcomes of this section.

### 2.1. Gut Microbiota as a Risk Factor of Acute Pancreatitis

As highlighted above, alterations in the gut microbiota can significantly influence the progression of AP. However, beyond understanding microbiota changes during the disease, recent research also suggests that composition of the gut microbiome may play a crucial role in the risk of developing AP, acting as a significant environmental factor influencing disease onset [48,49,50].

Several studies have provided valuable insights into the microbial populations associated with an increased or decreased risk of pancreatitis [51]. In particular, Zhou et al. demonstrated that the presence of Bacteroidales and the class of Bacteroidia were linked to an increased risk of developing pancreatitis [51]. Conversely, beneficial genera such as *Coprococcus* and *Eubacterium fissicatena* were found to exert protective effects and genera like *Prevotella*, *Ruminiclostridium*, and *Ruminococcaceae* were considered protective factors against pancreatitis. Notably, a positive correlation with the phylum Proteobacteria and genus *Lachnospiraceae* was identified as a risk factor for AP, whereas *Holdemania* was inversely correlated with the disease [51]. To further clarify these results, Qu et al. discovered that an elevated relative abundance of *Bacteroides plebeius* was significantly correlated with an increased risk of AP [52]. Similarly, Zhu et al. conducted a randomized controlled trial that highlighted the potential of genetic prediction for certain genera (*Eggerthella*, *Erysipelatoclostridium*, *Flavonifractor*, *Methanobrevibacter*, *Prevotella*) in reducing the risk of developing AP [53]. In contrast, genera such as *Eubacterium eligens*, *Coprococcus*, and *Haemophilus* were associated with an elevated risk of AP [53]. The most recent bidirectional Mendelian randomization analysis conducted by Nan et al. further corroborated these findings, identifying Firmicutes, *Erysipelatoclostridium*, *Flavonifractor*, *Methanobrevibacter*, and *Prevotella* as protective against AP, while *Eubacterium eligens* and related genera contributed to increased risk [54]. Despite the valuable insights these studies have provided, most of them have relied on fecal samples, which may not fully capture the complete microbiome within the gastrointestinal tract. To address this limitation, the use of mucosal samples is recommended for future investigations [55]. Moreover, the gut microbiome encompasses not only bacteria but also fungi and viruses, suggesting that a more comprehensive approach, including mycobiome analysis, could provide an even deeper understanding of how the microbiota influences the risk of AP [55]. As our understanding of the microbiome expands, these insights may open new avenues for predictive diagnostics, preventative strategies, and targeted therapies aimed at reducing the risk of acute pancreatitis and improving patient outcomes.

### 2.2. Microbiota Differences Based on AP Etiology

The gut microbiota composition in AP can differ depending on its underlying cause, with specific microbial shifts observed in various etiologies such as gallstones, alcohol abuse, and hyperlipidemia [41]. Liu et al. observed that most of the bacterial species identified as more abundant in acute pancreatitis patients were present in cases of acute biliary pancreatitis, with the notable exceptions of *Veillonella dispar* and *Christensenella minuta*. Additionally, *Streptococcus infantis* was found to be uniquely more abundant in patients with acute biliary pancreatitis, a pattern not observed in other causes of pancreatitis [41]. Additionally, *Bilophila wadsworthia* was significantly more abundant in both acute hyperlipidemic and biliary pancreatitis, with its levels positively correlating with triglyceride concentrations [41]. Previous studies have shown that *B. wadsworthia*, a predominant sulfidogenic bacterium, thrives on dietary lipids and may exacerbate intestinal barrier dysfunction and bile acid dysmetabolism, which in turn contributes to systemic inflammation [56]. This evidence supports the hypothesis that *B. wadsworthia* plays a role in the pathogenesis of both acute hyperlipidemic and biliary pancreatitis [41]. Additionally, species like *Eubacterium eligens*, which were depleted in acute pancreatitis (AP), showed a negative correlation with both the severity of pancreatitis and various triglyceride levels [41]. This suggests that these bacteria may play a role in regulating triglyceride metabolism, which is implicated in the progression of AP [57,58,59]. In more detail, in patients with hypertriglyceridemia-induced pancreatitis (HTGP), gut microbiota shows a distinct pattern compared to other forms of pancreatitis [60,61]. These patients exhibit reduced gut microbiota abundance and diversity. Specifically, there is an increase in the abundance of *Escherichia–Shigella* and *Enterococcus*, while the levels of *Dorea longicatena*, *Blautia wexlerae*, *Faecalibacterium*, and *Bacteroides ovatus* are decreased [62,63]. Li et al. further observed a higher relative abundance of Enterococcaceae in HTGP patients, while *Escherichia–Shigella* and *Bacteroides* were less abundant [64]. Notably, the abundance of *Faecalibacterium* and *Bacteroides uniformis* was inversely correlated with the severity of the disease [65]. A key aspect of HTGP pathogenesis is the interaction between gut microbial metabolites and immune regulation. *B. uniformis* has been shown to increase the production of taurine in the gut and its systemic levels. Taurine helps maintain neutrophil redox balance and limits inflammation by regulating cytokine release [66,67]. This highlights the significant role of gut microbial metabolites in modulating immune responses and suggests that *B. uniformis* plays a protective role by preventing pancreatic injury and reducing systemic inflammation in HTGP [68,69]. Notable changes in gut microbiota composition are also observed in alcohol-associated pancreatitis. Philips et al. found that patients with acute alcoholic pancreatitis had higher levels of Actinobacteria and lower levels of Bacteroidetes [68]. In contrast, Ciocan et al. reported that patients with chronic alcoholic pancreatitis had elevated levels of intestinal Proteobacteria and decreased levels of Bacteroidetes [69]. Additionally, at the genus level, *Klebsiella pneumoniae*, *Lactobacillus*, *Enterococcus*, and *Sphingomonas* were more prevalent in patients with chronic alcoholic pancreatitis than in those with the acute form of the disease [69]. Alcohol consumption can damage the intestinal barrier, leading to increased intestinal permeability. This occurs through the extraction and dissolution of intestinal mucosal lipids, which decreases the hydrophobicity of the mucosal surface [70]. This hydrophobicity is an important aspect of the intestinal barrier function and serves as a marker for it [70]. Mutlu et al. found that serum endotoxin levels were significantly higher in alcoholic individuals (with or without liver damage) than in healthy controls [71]. The key factor influencing microbiota composition appears to be chronic alcohol consumption, rather than liver disease [71]. This suggests that alcohol consumption causes a “double-hit” mechanism of damage: direct damage to the pancreatic tissue and indirect damage due to increased intestinal permeability, which can lead to secondary infections in acute pancreatitis. Table 2 summarizes the microbiota alterations observed in AP based on different etiologies.

### 2.3. Altered Microbial Communities and Disease Severity

The composition of the gut microbiota changes significantly with the severity of acute pancreatitis, suggesting a potential role in determining disease progression. Li et al. demonstrated an inverse correlation between the prevalence of *Faecalibacterium* and *Bacteroides uniformis* and disease severity, indicating that specific bacterial profiles influence the clinical course [62]. Liu et al. observed that species such as *Eubacterium eligens*, depleted in AP, negatively correlated with disease severity and serum triglyceride (TG) levels, suggesting a role in AP progression through TG metabolism regulation [41]. Moreover, Tan et al. found a marked reduction in dominant bacterial diversity in patients with moderately severe acute pancreatitis (MSAP) and SAP compared to healthy controls, despite no significant changes in total bacterial count [35]. In MSAP and SAP groups, populations of Enterobacteriaceae and *Enterococcus* increased, while *Bifidobacterium* levels were significantly lower. Ammer-Herrmenau et al. revealed no significant differences in α-diversity and β-diversity of buccal and rectal microbiota within 72 h of admission [24]; however, Bray–Curtis analysis identified a distinct microbial signature in severe AP (RAC III), with enriched species belonging to genera such as *Parabacteroides distasonis*, *Enterocloster bolteae*, and *Roseburia hominis*, known SCFA producers. Furthermore, Hu et al. identified distinct microbiota changes in patients with AP-associated acute respiratory distress syndrome (ARDS), including elevated levels of the Proteobacteria phylum, Enterobacteriaceae family, *Escherichia–Shigella* genus, and *Klebsiella pneumoniae* [72]. The AP non-ARDS group showed reduced *Bifidobacterium* levels. Notably, random forest modeling highlighted the *Escherichia–Shigella* genus as a predictor for ARDS development in AP patients [72]. Zhu et al. demonstrated that opportunistic pathogens like Enterobacteriaceae and *Enterococcus* were significantly elevated in SAP patients, whereas beneficial bacteria, such as Bifidobacteriaceae, were notably diminished compared to the MAP group [73]. A reduction in microbial diversity and increased Enterobacteriaceae levels were also observed in patients with infectious acute necrotising pancreatitis (ANP) relative to non-ANP groups, alongside lower levels of *Clostridium* and Bacteroidetes [74]. Notably, *Enterococcus faecalis* and *Finegoldia magna* emerged as potential indicators of infected pancreatic necrosis and disease severity [74]. The detection of *Enterococcus* in both drainage fluid and peripheral blood of SAP patients underscores the role of bacterial translocation from the gastrointestinal tract to the pancreas and circulatory system in exacerbating disease [75,76]. Lastly, Wang et al. analyzed the gut microbiota of MAP and SAP patients across different stages of the disease (first and second weeks) and found that SAP patients exhibited significantly reduced microbial diversity [77]. This included an increase in pathogenic bacteria such as Proteobacteria and a decrease in beneficial species like *Blautia*, *Enterococcus*, *Faecalicatena contorta*, and *Ruminococcaceae*. By the second week, the SAP group showed a marked rise in pathogens such as *Stenotrophomonas* and *Enterobacter*, while beneficial bacteria like *Blautia* and *Christensenella* further declined [77]. These findings reveal a progressive shift toward a pathogenic gut microbiota profile in SAP, emphasizing its potential impact on disease worsening and systemic complications. Table 3 summarizes the microbiota alterations observed in AP based on disease severity.

### 2.4. Gut Barrier Alteration

Alterations of intestinal permeability represent a significant feature of AP, driven by microcirculatory disturbances such as hypovolemia, splenic vasoconstriction, and fluid loss into the “third space” [78,79]. Nearly 59% of AP patients exhibit disrupted intestinal barriers, leading to increased permeability, as shown by Capurso et al. [80]. This facilitates the translocation of gut microorganisms, toxins, and inflammatory cytokines into the portal circulation, potentially aggravating pancreatic injury and contributing to SIRS [80]. The gut itself has been described as an “undrained abscess”, reflecting its role as a reservoir for pathogenic bacteria and inflammatory mediators that are not effectively cleared. This stagnant pool of harmful agents can perpetuate inflammation, sepsis, and multiorgan failure (MOF) in conditions like SAP [81,82,83]. A key factor in intestinal barrier integrity is the production of SCFAs, such as butyrate by gut microbiota, which regulate tight junction proteins (e.g., claudin−1, ZO−1, and occludin), by increasing their expression and reinforcing mechanical, mucosal, and chemical barriers [84,85,86,87,88,89,90]. During AP, there is a marked reduction in SCFA-producing bacteria, exacerbating intestinal barrier dysfunction [91]. In severe cases, early alterations in permeability are linked to endotoxin translocation, systemic endotoxemia, and increased susceptibility to MOF and mortality. Studies in humans and animal models corroborate these findings. For instance, elevated levels of biomarkers such as diamine oxidase (DAO) and D-lactate, released when the intestinal barrier is damaged, indicate heightened intestinal injury during AP [91]. In patients with SAP, Ammori et al. observed a significant increase in intestinal permeability to macromolecules, specifically polyethylene glycol 3350, compared to those with mild attacks and healthy controls [92]. No changes in permeability were found in patients with mild AP. These alterations in intestinal permeability appeared within 72 h of disease onset and strongly correlated with clinical outcomes [93]. In fact, patients who developed multiorgan failure (MOF) or died exhibited more pronounced changes in permeability. Further supporting these findings, Juvonen and colleagues demonstrated that patients with severe AP had a significant increase in gut permeability to micromolecules (sugar probes) within 48 h of hospital admission [93] compared to those with mild attacks. Tan et al. showed similar patterns of gut microbiota dysbiosis in both severe and mild AP patients, suggesting that gut microbiota imbalances may precede the development of MOF and SIRS [35]. This dysbiosis could worsen the inflammatory response, making patients more susceptible to infections and organ failure, reflecting the critical connection between intestinal barrier function, and the systemic complications of AP [80,94].

## 3. Potential Microbiota-Based Therapeutic Target for AP

**Figure 2:**
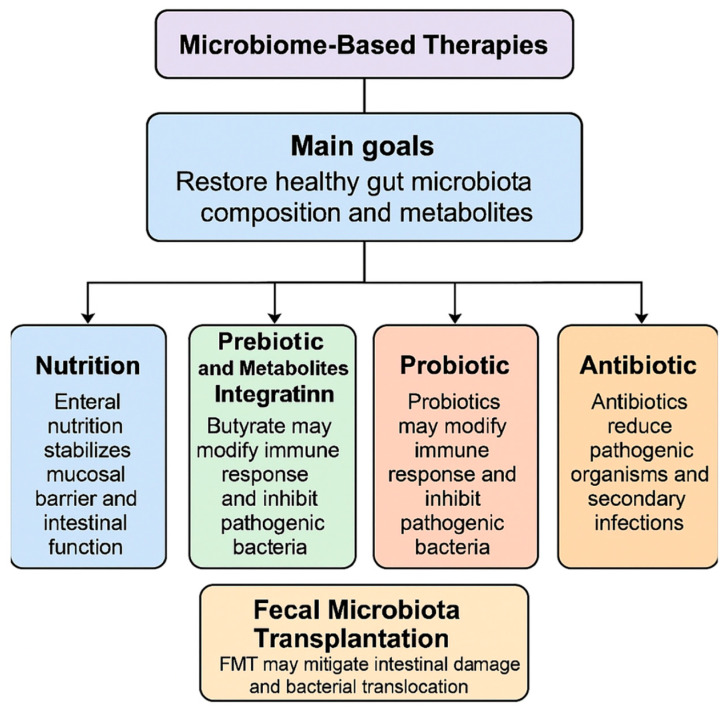
Outcomes targeted by the different microbiome-based therapies.

The primary goals of microbiome-based therapies, which include probiotics, prebiotics, antibiotics, FMT, and enteral nutrition, are to alter the composition of the gut microbial community and the amount of metabolites derived from the microbiota [95]. By resetting the entire flora or supplementing with certain beneficial organisms and their byproducts, these therapeutic approaches aim to eradicate harmful microorganisms, reducing inflammation and avoiding bacterial translocation [95]. Key data and outcomes of this section are summarized in Figure 2.

### 3.1. Nutrition

Enteral nutrition is one of the key therapeutic interventions applied during AP [95]. There is clear evidence, as stated in guidelines regarding treatment of AP, that enteral nutrition is far superior if compared to parenteral nutrition in reducing mortality, complication rate and hospital stay [95]. This is particularly evident when the enteral nutrition is administered during the first 48 h [96,97]. The rationale behind its widespread use is its regulatory effect on gut microbiota, which stabilizes the mucosal barrier and intestinal function, while parenteral feeding is reserved for patients who cannot tolerate oral or enteral feeding [98]. Specific substances such as glutamine, arginine, and n−3 fatty acids may be responsible of these effects, as demonstrated in murine models [99]. Evidence presented by Huang et al. demonstrates that enteral administration of glutamine enhances intestinal barrier function by mitigating intestinal permeability during the early stages of AP [100]. Moreover, a 2016 meta-analysis revealed that nutritional support provided by the administration of glutamine was associated with an increase of albumin levels and a reduction in C-reactive protein levels, and a lower incidence of infectious complications, especially if administered parenterally [101]. This effect may be attributed to glutamine’s critical role in supporting the normal function of gut-associated lymphoid tissue (GALT) and its involvement in the synthesis of glutathione, a key antioxidant.

### 3.2. Prebiotic and Metabolites Integration

The administration of metabolites and prebiotics molecules represents a potential avenue for the treatment of SAP. Butyrate supplementation has been demonstrated to reduce mortality and alleviate pancreatic injury in SAP mice [37]. This is achieved by strengthening the gut barrier and reducing bacterial translocation as stated previously [37]. A large preclinical study has demonstrated that the administration of parenteral therapy with n−3 fatty acids has the effect of reducing the histopathologic severity of acute necrotizing pancreatitis. This effect has been attributed to the early inhibition of prostaglandin, such as Prostaglandin E2 (PGE2) and Prostaglandin F1-alpha (PGF1α), and reduction of lipid peroxidation [102]. Moreover, Butyric acid has been demonstrated to reduce systemic inflammation by inhibiting the STAT1/AP1 signaling pathways in peritoneal macrophages, as well as the presence of NLRP3 inflammasomes and pro-inflammatory factors [103]. In a similar way, acetate reduce the inflammatory response of the organism thanks to its antimicrobial, anti-inflammatory, and antioxidant properties. Moreover, valproic acid is capable of attenuating AP by reducing myeloperoxidase activity and the local tissue damage to other target organs [104]. Nevertheless, treatment of AP by parenteral supplementation of metabolites/prebiotic has primarily been investigated in animal models, with no evidence available from human studies.

### 3.3. Probiotic

Probiotic-based therapies proved to be potentially effective in animal models of AP [105]. However, their role in human disease is still unclear. Four potential mechanisms by which probiotics may affect human health are currently being investigated: (1) local and systemic immunomodulatory effects, mediated by specific strains of probiotics [96]; (2) production of metabolic byproducts, known as postbiotics [106], capable of influencing intestinal homeostasis (e.g., Poly-Unsatured Fatty Acids (PUFAs), SCFA, Glutammine) and regulating the enteric nervous system; (3) reversing inflammatory change or strengthening the gut barrier through endogenous pathways (e.g., defensine production, mucus layer [107,108]; and (4) indirect inhibition or direct eradication of pathogenic bacteria [109]. Rycther et al. showed that the administration of multispecies probiotics for a period of two days prior to the onset of AP effectively reversed the disruption of intestinal barrier function that occurs in the late phase of AP in a mouse mode [79]. However, the same treatment has not demonstrated comparable beneficial effects when administered during AP [79]. Furthermore, the efficacy of probiotics, either as a monotherapy or in combination with two antibiotics, was investigated in murine models with induced AP. Probiotics were found to reduce the translocation of pathogenic bacteria, while the combination of probiotics and antibiotics led to a reduction in histopathological scores (which included edema, inflammatory infiltration, fat necrosis, parenchymal necrosis, and hemorrhage) and oxidative markers. These findings suggest that the combined use of probiotics and antibiotics may be a more effective approach for mitigating pancreatic damage compared to probiotics alone [110]. Meanwhile in clinical studies the use of probiotics in AP showed mixed results. Wan et al., in a randomized, double-blind study, explored the potential of probiotics to reduce hospital stay duration in patients with mild acute pancreatitis [111]. These findings suggest that probiotic supplementation may effectively and significantly shorten hospital stays for patients with mild acute pancreatitis, as it was associated with a faster resolution of abdominal pain and an earlier transition to successful oral feeding than in the placebo group. However, several critical considerations must be considered. Firstly, there are various types of probiotics, and their therapeutic effects may differ. Additionally, it is important to consider that adverse reactions may vary significantly, particularly in patients with SAP As a matter of fact, the PROPATRIA trial (Probiotics Prophylaxis in Patients with Predicted Severe Acute Pancreatitis), a multicenter large-scale randomized controlled trial (RCT), aimed to determine whether the enteral administration of a multispecies probiotic preparation could reduce infectious complications in patients with predicted SAP [112]. However, the trial was terminated early after findings revealed that probiotic combination treatment was ineffective in reducing the incidence of infectious complications in SAP patients [112]. Furthermore, the study indicated that probiotic supplementation might have increased the risk of mortality, leading to the premature discontinuation of the trial [112]. This finding regarding SAP and probiotics was later confirmed by a systematic meta-analysis conducted by Gao et al., which showed that, compared to control groups (standard care without probiotics), probiotics did not result in a statistically significant reduction in mortality rates or the risk of organ failure [34]. However, they did result in a notable reduction in hospital stays, with a weighted mean difference of around five days [34]. The existing literature on the use of probiotics in SAP and critically ill patients is limited by several factors [113], including the variability in probiotic strains, dosages, and durations of administration, as well as the relatively small sample sizes used in these studies. Although probiotics are generally well tolerated with rare adverse events, the findings of the PROPATRIA trial underscore the need for stringent safety monitoring. Further research aimed at identifying the optimal characteristics of effective probiotics, along with a deeper understanding of their mechanisms of action, will help clarify their true beneficial effects in acute pancreatitis, particularly in mild cases where they appear to have a positive impact.

### 3.4. Antibiotic

The administration of antibiotics has been demonstrated to impede the proliferation of pathogenic organisms in patients with SAP, thereby reducing the incidence of secondary infections and their associated complications [114]. Nevertheless, the excessive use of broad-spectrum antibiotics has led to the emergence of multidrug-resistant strains and increased mortality rates in mouse models of AP [114]. In a mouse model study, a combination of antibiotics—specifically vancomycin, neomycin, and polymyxin B—was shown to prevent the development of severe experimental AP. This observation suggests that selectively targeting specific bacterial populations may offer a promising therapeutic strategy for the management of AP, potentially reducing the severity of the condition [115]. An alternative approach involving selective digestive decontaminants (SDD) has been proposed to modify the composition of the microbiota without relying on broad-spectrum antibiotics, thereby avoiding the issue of antibiotic resistance. This strategy has been tested in both patients with SAP and in mouse models, with the aim of reducing the risk of infection while preserving the beneficial microbial populations [37,116]. However, in the human population, although the use of SDD indicated a positive trend in reducing complications and mortality associated with SAP, the results of the study did not achieve statistical significance. This suggests that while SDD may offer potential benefits, further investigation with larger sample sizes or more refined methodologies may be needed to confirm its efficacy [116]. Current guidelines for treatment of acute AP recommend the use of antibiotics only when there is evidence or a strong suspicion of infection [95]. One of the primary reasons for this recommendation is the growing concern over antibiotic resistance. Consequently, we propose that targeting specific pancreatic microbiota or pathogenic gut bacteria with narrow-spectrum antibiotics, in combination with SDD, could represent a promising area of research. This approach may offer a safer alternative that reduces the risk of developing antibiotic resistance while still targeting the underlying bacterial threats.

### 3.5. Role of Fecal Microbiota Transplantation in AP

Fecal microbial transplantation (FMT) represents a therapeutic strategy whereby fecal material from healthy donors is introduced into the patient’s gut to restore gut dysbiosis. This approach is currently being used as a therapeutic strategy for a number of diseases, for example, hepatic encephalopathy [117], IBS, [118] and, in particular, in recurrent C. difficile infections [119]. However, its role as a potential treatment in acute pancreatitis has not been clarified. Yu et al. observed that FMT significantly mitigated intestinal mucosal damage in AP mice, as evidenced by a reduction in inflammatory cell infiltration and an increase in secretory IgA concentrations [120]. Conversely, the findings of Berg et al. indicated that FMT resulted in a notable elevation in pathogenic bacterial translocation and mortality in AP mice [37]. A recent randomized, single-blind study investigated whether fecal microbiota transplantation decreases intra-abdominal pressure and improves gastrointestinal dysfunction and infective complications in acute pancreatitis [121]. This study did not show any significant difference between the FMT and the placebo group even though its statistical sample was not numerous. Due to this inconclusive finding, a promising direction for future research would be to investigate the alterations in gut microbiota in patients with acute pancreatitis (AP) and identify beneficial bacterial strains. This approach could facilitate the optimization and standardization of FMT, enabling a more comprehensive evaluation of its potential role in the treatment of AP.

## 4. Experience of Microbiome Modulation in Other Acute Settings

**Figure 3:**
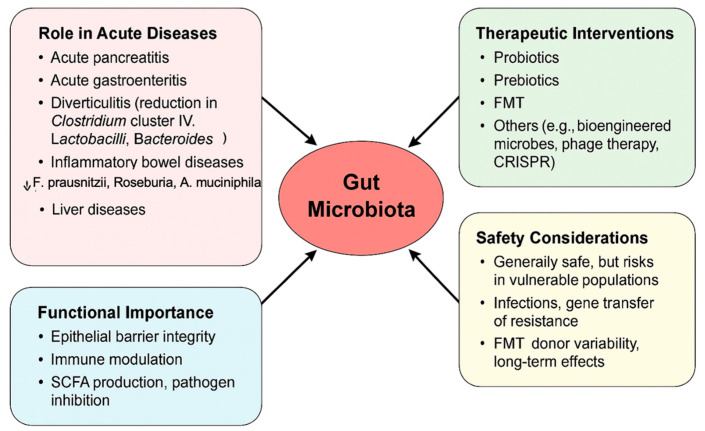
Main findings on gut microbiota and other gastrointestinal diseases, and safety considerations.

Gut microbiota may influence acute diseases. Key data and outcomes of this section are summarized in Figure 3. The alteration of gut microbiota composition (dysbiosis) may be responsible or exacerbate not only acute pancreatitis but also some other acute diseases as acute gastroenteritis [122], diverticulitis [123], inflammatory bowel diseases [124], and liver diseases [125]. For example, in acute diverticulitis has been observed a reduction of taxa as *Clostridium cluster IV*, *Lactobacilli* and *Bacteroides* [123]. In inflammatory bowel disease, *Roseburia*, *Phascolarctobacterium* and *Faecalibacterium prausnitzii* were significantly decreased, while *Clostridium* was increased [126]. In ulcerative colitis *Akkermansia muciniphila* decreased while adherent-invasive *Escherichia coli*, *Clostridium difficile*, some *Salmonella* and *Helicobacter* species increased [127]. As regards liver diseases (as non-alcoholic fatty liver disease), an increased abundance of species *Clostridium*, *Anaerobacter*, *Streptococcus*, *Escherichia coli*, *Ruminococcus* and *Lactobacillus* have been reported, whereas *Oscillibacter*, *Flavonifaractor*, *Odoribacter* are less abundant [128]. It is well known that numerous species harboring the human gut play a key role in protective, structural, and metabolic functions. They can act as a barrier against pathogens, thus maintaining the integrity of the gut epithelial barrier, influencing the tight junction permeability, and secreting antimicrobial substances [129]. They can also modulate and activate innate and adaptive immune system, enhancing cytokine production, produce SCFAs, and ferment non-digestible substrates and mucus, maintaining a healthy intestinal epithelium [129]. Literature studies about acute gastrointestinal disorders as acute gastroenteritis, colitis, diverticulitis, and liver diseases have observed a persistent gut inflammation and permeability in case of failure to restore a balanced composition of gut microbiota species. The intervention to modulate the gut microbiome can range from the use of prebiotic, probiotics, symbiotic, postbiotics, and fecal microbiota transplantation to the more complex technology with bio-engineered commensals, or drugs targeting selected-microbial proteins and metabolites, phage therapy, Clustered Regularly Interspaced Short Palindromic Repeats (CRISPR)-Cas9-based therapy to treat and reverse acute diseases [130]. The research on probiotics has made significant progresses. The use of *Lactobacillus*, *Bifidobacterium*, and *Saccharomyces* proved effective and safe in many acute gastrointestinal diseases. In addition, new probiotics strains, such as *Faecalibacterium prausnitzi*, *Akkermansia muciniphila*, and several *Clostridia*, are under investigation. Probiotics have shown beneficial effects in restoring the balance of gut microbiota communities, in preventing pathogens from colonization, and in maintaining the integrity of epithelial barrier. Furthermore, probiotics resulted in effective modulating of the immune system [131]. They can increase the number of macrophages and dendritic cells in the intestinal lamina propria, driving the synthesis of cytokines and participating in the signaling cascades, involving the Toll-like receptor 2 and nuclear factor kB. In addition, probiotics could contribute to regulate T helper 17 cells, regulatory T cells, and gut-specific B cells. Furthermore, probiotics can secrete propionic acid and butyric and acetic acid, which are considered antimicrobial compounds able to inhibit activity against pathogens [131]. In acute diverticulitis, probiotics (i.e., *Lactobacillus reuteri*) proved effective in decreasing inflammation, stimulating a correct bowl motility, and preventing the development of complications [132,133,134,135,136]. In liver diseases such as cirrhosis, probiotics have shown anti-inflammatory and anti-fibrotic effects and an improvement of liver functions [137]. Furthermore, researchers suggest a role for probiotics in preventing post-transplant infections and in improving hepatic encephalopathy. The only concern is about sepsis and critically ill patients with advanced liver disease, in which they are not still recommended [137]. In inflammatory bowel diseases, probiotics such as *E. coli Nissle* have proved safe and effective in maintaining ulcerative colitis in remission, and *Bifidobacterium* and VSL#3 for induction of remission in case of active mild-to-moderately ulcerative colitis (mechanisms include the reduction of oxidative stress, the modulation of intestinal immune response, the repair of the gut barrier, etc.) [138]. Currently, there are not definitive conclusions as regards the use of probiotics in Crone’s disease. Research into the very complex gut microbiota, which includes also bacteriophages, eukaryotic virus, funghi, and archea, is an open field in continuing expansion. How to modulate the microbiota and its interaction with the host is an exciting challenge for researchers, where it is useful to add something new in the prevention or treatment of common human acute and chronic diseases.

### 4.1. Safety of Microbiota-Based Therapy

Although treatments with microbiota-based therapies, including probiotics and FMT, show promising results, safety concerns have been reported in the literature in certain population subgroups. Indeed, while restoring the gut microbiome, safety profiles of these treatments need caution to avoid adverse effects in the human host.

Probiotics are nowadays widely used and are generally considered safe in healthy individuals, in whom potential benefits have been described for gastrointestinal and metabolic disorders, and also in mental health. However, vulnerable populations in this setting are neonates with low birth weight, critically ill patients, or individuals with compromised immune systems. Indeed, case reports and clinical trials have underlined the need for caution, particularly when prescribing probiotics to high-risk populations [139,140]. The adverse events reported in such groups include sepsis, fungemia, and gastrointestinal ischemia, which have been linked to certain strains like *Lactobacillus rhamnosus GG* and *Saccharomyces boulardii* [141,142].

However, adverse events are not limited to infection, as the issue of possible horizontal gene transfer of antibiotic resistance genes has been reported in experimental models. It has been demonstrated that some probiotic strains are able to transfer resistance genes to pathogenic bacteria, which raises concerns about the wider implications for antimicrobial resistance [141,142].

In addition, two systematic reviews of large-scale probiotic trials came to the same conclusion: adverse events and safety are poorly reported. This again points to the need for independent, high-quality trials testing efficacy and risks, especially in at-risk populations [139,143].

FMT is the process of transferring fecal material from a healthy donor to a recipient to reestablish an appropriate microbiota. The most common indications nowadays regard recurrent infections caused by *Clostridioides difficile*, on which FMT has proven efficacy and safety. However, this practice carries some risks, too. Potential complications include the transmission of pathogens, adverse immune responses, and even unknown long-term effects on health [144].

Common adverse reactions include gastrointestinal symptoms like diarrhea, abdominal pain, and flatulence. Rare but serious complications reported include systemic infections and autoimmune reactions [145,146].

Therefore, strict screening among donors is conducted to lessen these risks; however, it is not possible to completely avoid the risk of transmission of unknown or antibiotic-resistant microorganisms [147].

Long-term effects of changes in the gut microbiome are not well understood. Possible changes in metabolism or immune function could result in other diseases not directly related to the disorder for which treatment was started, such as obesity or autoimmune diseases [144,148].

### 4.2. Standardization Issues

Currently, the lack of standardization in microbiota-based therapies creates significant challenges for their clinical application and regulatory approval. The variability of definitions, different production, and evaluation methodologies further complicates the development of universally accepted guidelines.

Probiotics often differ significantly in composition and quality, even within the same declared strains, due to inadequate strain characterization and discrepancies in labeling and manufacturing processes. Some products fail to uphold the specified viable microorganism count until their expiration date, which can hinder their effectiveness [140,149]. Additionally, the lack of consensus regarding a minimum effective dose for various indications poses a challenge. The effectiveness of probiotics is also influenced by some factors such as individual gut microbiota differences, dietary habits, and concurrent medications. These variations highlight the importance of tailoring probiotic therapy to individual needs [141,143]. Concerning FMT, most of the issues in standardization are related to donor selection and screening. There is great variability in the microbial composition of fecal material between different donors, and there is no consensus on which strains are required for therapeutic efficacy. Further complicating this process, there is a lack of standardization for donor screening, preparation, and storage [146,150].

The route of administration such as capsule, colonoscopy, or enema is another variable that would affect the outcome. However, there is no consensus on the optimal method for various conditions, making it challenging to establish uniform guidelines [145].

Dosing regimens have not been standardized. Indeed, the amount and frequency of FMT to achieve therapeutic success vary greatly. While personalized approaches may increase their efficacy, they introduce even more complicating factors into the process of the standardization [147].

Regulation of microbiota-based therapies differs substantially across the world: while FMT is considered to be a drug in some countries, it is regarded as a procedure in others, leading to inconsistencies in its application and regulation [151].

The establishment of stool banks and standardized manufacturing protocols reflects the development in quality control. For example, organizations like OpenBiome have developed policies on donor screening, preparation, and storage of materials with the aim of optimizing safety in this context [145,146].

## 5. Challenges and Perspectives

Despite a great scientific interest in the role of gut microbiota and probiotics in pancreatic diseases, the path towards an effective clinical integration of this knowledge still shows many obstacles and open questions. One of the main challenges is the need to establish clear and commonly shared guidelines, together with the creation of stool banks that are strictly controlled from the healthcare and qualitative points of view. With high standards in terms of safety and traceability, it might be possible to achieve and warrant reliable and replicable treatments.

Personalized therapies based on the microbiome might greatly improve management of AP and, to do so, new technologies such as metagenomics, metabolomics, bioengineered probiotics, and genomic editing via CRISPR-Cas9 open further routes to understand and modulate complex gut microbiota-host interactions.

## 6. Conclusions

In conclusion, the gut–pancreas axis, shaped by microbial metabolites, immune signaling, and neuroendocrine interactions, represents a crucial regulatory mechanism in AP pathophysiology and in disease progression, contributing to increased intestinal permeability, systemic inflammation, and disease severity. Microbiota-targeted therapies, including probiotics, prebiotics, enteral nutrition, antibiotics, and FMT, offer promising strategies to reduce inflammation and preserve gut integrity by modulating gut microbial composition and function. While experimental studies support these approaches, clinical outcomes remain inconsistent, underscoring the need for rigorous trials to optimize efficacy and safety. Despite their potential, microbiota-based treatments pose challenges, particularly regarding safety in critically ill patients. Probiotic efficacy varies by strain, disease severity, and patient-specific factors, while the lack of standardized protocols for probiotics and FMT limits clinical reliability. Establishing clear guidelines and quality-controlled stool banks is essential for ensuring safety and effectiveness. Future research should focus on personalized microbiome-based therapies, leveraging advanced technologies like metagenomics, metabolomics, bioengineered probiotics, and CRISPR-Cas9. Unraveling the intricate gut–host interactions will be crucial for developing targeted, safe, and effective microbiota-directed treatments for AP. By leveraging precision medicine, microbiome research has the potential to transform AP management, improving patient outcomes and shaping novel therapeutic strategies for inflammatory diseases.

## Figures and Tables

**Table 1:** Microbiota alterations observed in AP.

Study	Type of Sample	Subject	Phylum Level	Family, Genus, or Species	Microbial Evaluation
Tan et al. (2015) [35]	Feces	AP patients vs. Healthy volunteers	-	↓*Bifidobacteria* ↑Enterobacteriaceae ↑*Enterococcus*	PCR-DGGE
Wahlström et al. (2016) [36]; Van den berg (2021) [37]; Almeida et al. (2019) [38]		AP patients vs. Healthy volunteers	↑Bacteroidetes ↑Proteobacteria ↓Firmicutes ↓Actinobacteria	↑*Escherichia-Shigella* ↑Erysipelotrichaecease ↑*Streptococcus* ↑*Enterococcus*	16S
Zhang et al. (2018) [39]	Feces	AP patients vs. Healthy volunteers	↑Bacteroidetes ↑Proteobacteria ↓Firmicutes ↓Actinobacteria	-	16S
Chen et al. (2017) [40]	Feces	AP patients vs. Healthy volunteers	↓Saccharibacteria ↓Tenericutes	↑*Escherichia-Shigella* ↑*Phascolarctobacterium* ↓*Candidatus Saccharimonas;* ↓Prevotellaceae ↓Lachnospiraceae ↓*Ruminiclostridium* ↓*Ruminococcaceae*	16S
Liu et al. (2024) [41]	Feces	AP patients vs. Healthy volunteers	-	↑*E. coli* ↑*Enterococcus* ↑*Parabacteroides* ↑*Clostridium* ↑*Veillonella*	16S
Zhao et al. (2023) [47]	Descending duodenum	MAP patients vs. Healthy individuals	-	↑*Streptococcus* ↑*Neisseria* ↓*Actinobacillus* ↓*Oribacterium*	16S

AP = acute pancreatitis, MAP = mild acute pancreatitis. PCR = polymerase chain reaction, DGGE = denaturing gradient gel electrophoresis. ↑ = increased levels, ↓ = reduced levels.

**Table 2:** Microbiota alterations observed in AP based on different etiologies.

Study	Type of Sample	Subject	Phylum Level	Family, Genus, or Species	Microbial Evaluation
Liu et al. (2024) [41]	Feces	Biliary AP vs. others AP etiologies	-	↑*Bilophila wadsworthia* ↑*Streptococcus infantis* ↓*Veillonella dispar* ↓*Christensenella minuta*	whole-metagenome shotgun sequencing
Liu et al. (2024) [41]	Feces	HTGP vs. others AP etiologies	-	↑*Bilophila wadsworthia*	whole-metagenome shotgun sequencing
Li et al. (2023) [61]	Feces	HTGP vs. Healthy volunteers	↑Firmicutes ↓Proteobacteria	↑Enterococcaceae ↓*Escherichia-shigella* ↓*Bacteroides* ↓*Faecalibacterium*	16S
Philips et al. (2019) [68]	Feces	Alcoholic AP vs. Healthy volunteers	↑Actinobacteria ↓Bacteroidetes	↑*Moraxella* ↑*Acinetobacter*	16S
Ciocan et al. (2018) [69]	Feces	Alcoholic AP vs. Alcoholic	↑Proteobacteria ↓Bacteroidetes	↑*Klebsiella pneumoniae* ↑*Lactobacillus* ↑*Enterococcus* ↑*Sphingomonas*	16S

HTGP = hypertriglyceridemia-induced pancreatitis. ↑ = increased levels, ↓ = reduced levels.

**Table 3:** Microbiota alterations observed in AP based on disease severity.

Study	Type of Sample	Subject	Phylum Level	Family, Genus, or Species	Microbial Evaluation
Ammer-herrmenau et al. (2024) [24]	Buccal and rectas swab	SAP vs. non-SAP	-	↑*Leuconostoc citreum* ↑*Rothia nasimurium* ↑*Leuconostoc pseudomesenteroides* ↑ *Clavibacter michiganensis* ↓*Streptococcus pyogenes* ↓*Lawsonella clevalandensis* ↓*Aerococcus unnae* ↓*Finegoldia magna* ↓*Streptococcus dysgalactiae* ↓*Streptococcus pseudoporcinus* ↓*Peptoniphilus harei* ↓*Anaerococcus mediterraneensis* ↓*Peptoniphilus ivorii*	
Tan et al. (2015) [35]	Feces	AP patients vs. Healthy volunteers	-	↑Enterobacteriaceae ↑*Enterococcus* ↓*Bifidobacterium* ↔*Lactobacillus*	PCR-DGGE
Liu et al. (2024) [41]	Feces	SAP patients vs. MAP patients	-	↑*Eubacterium eligens*	whole-metagenome shotgun sequencing
Li et al. (2023) [61]	Feces	HTGP vs. Healthy volunteers	-	↓*Faecalibacterium* ↓*Bacteroides uniformis*	16S
Hu et al. (2023) [72]	Feces	ARDS patients vs. non-ARDS patients	↑Proteobacteria	↑*Escherichia-shigella* ↑Enterobacteriaceae ↑*Klebsiella pneumoniae* ↓*Bifidobacterium*	16S
Zhu et al. (2019) [73]	Feces	SAP patients vs. MAP patients	-	↑Enterobacteriaceae ↑*Enterococcus* ↓Bifidobacteriaceae	16S
Zou et al. (2022) [74]	Feces and rectal swabs	ANP patients vs. non-ANP patients	↓Bacteroidetes	↑Enterobacteriaceae ↑*Enterococcus faecalis* ↑*Finegoldia magna* ↓*Clostridium*	16S
Wang et al. (2024) [77]		SAP patients vs. MAP patients	↑Proteobacteria	↑*Stenotrophomonas* ↑*Enterobacter* ↓ *Blautia* ↓*Enterococcus* ↓*Faecalibacter contorta* ↓*Ruminococcaceae* ↓*Christensenella*	16S

SAP = severe acute pancreatitis, ANP = acute necrotising pancreatitis, ARDS = acute respiratory distress syndrome, ↔ = same levels. ↑ = increased levels, ↓ = reduced levels.
